# Amende

**Published:** 1853-03

**Authors:** 


*Amende.*—The address by Dr. Cotting, entitled *Nature in Disease*, contained in the February No. of this Journal, should have been credited to the *Boston Medical and Surgical Journal.* This credit was inadvertently omitted.

The short article in the same No. entitled "The Castor Bean Epidemic," was incorrectly credited to the Boston Med. and Surg. Journal. It should have been credited to the *North-Western Med. and Surg. Journal.*

